# Safety Lamps

**Published:** 1873-04

**Authors:** 


### SAFETY LAMPS.

Petroleum is so cheap and gives such a beautiful light, that it will continue to be used, in spite of its dangerous nature. One plan, previously noticed, is to fill a saucer half full of cold water, then pour on a tablespoonful or two of the oil; if flame applied to it sets it on fire, it is not safe to use it in lamps.

Put some oil in a tin cup, introduce a thermometer, apply heat to the bottom of the cup until it indicates one hundred degrees of Farenheit—if a hundred and five, it is still better; if on the application of a flame it does not flash, then it is a safe oil.

